# Life from death: ethical implications of uterus transplantation from deceased donors in global health

**DOI:** 10.1136/bmjgh-2024-018216

**Published:** 2025-06-19

**Authors:** Halina Suwalowska, Michael Parker, Adamu Addissie, Joseph Ali, David Bukusi, Abhilasha Karkey, Deborah Nyirenda, Srdjan Saso, Patricia Kingori

**Affiliations:** 1 University of Oxford, Oxford, UK; 2 School of Public Health, Addis Ababa University, Addis Ababa, Ethiopia; 3 Johns Hopkins University, Baltimore, Maryland, USA; 4 University of Nairobi, Nairobi, Kenya; 5 Oxford University Clinical Research Unit, Kathmandu, Nepal; 6 Community Engagement & Bioethics, Malawi-Liverpool-Wellcome Trust Clinical Research Programme, Blantyre, Malawi; 7 Liverpool School of Tropical Medicine, Liverpool, UK; 8 Imperial College Healthcare NHS Trust, London, UK; 9 Department of Population Health, University of Oxford, Oxford, UK

**Keywords:** Global Health, Health policies and all other topics

**SUMMARY BOX**

- Uterus transplantation (UTx) from deceased donors is a novel reproductive technology that has led to successful births globally. While ethically preferable to living donor UTx in avoiding donor harm, it raises complex ethical, social and policy challenges, particularly regarding consent, healthcare access and global inequalities.
- This article highlights the ethical implications of deceased donor UTx in a global context, examining issues of consent, the potential for reproductive tourism and socioeconomic disparities. It underscores the need for culturally sensitive ethical guidelines and a broader understanding of donor families' experiences.
- Findings from this study call for empirical research on donor families’ perspectives, equitable access to UTx and safeguards against exploitation. Policy makers and global health stakeholders must develop inclusive ethical frameworks to prevent reproductive tourism, ensure informed consent and protect marginalised populations.

In 2018, *The Lancet* reported that Brazilian researchers had successfully performed a groundbreaking uterus transplantation (UTx) from a deceased donor, leading to the birth of a healthy baby. This procedure involved removing a uterus from a 45-year-old deceased donor and transplanting it into a 32-year-old recipient, who was born without a uterus due to a congenital disorder.^1^ Since this pioneering achievement, UTx from deceased donors has gained global traction, offering a posthumous gift of life and fertility. By 2023, approximately 90 UTx procedures had been performed worldwide—both from deceased and living donors—with a growing number of using deceased donors, leading to over 45 successful births.^2^ For individuals affected by absolute uterine factor infertility—approximately 1 in 500 women of childbearing age globally^2^—UTx from deceased donors represents a vital reproductive option, particularly for those unable to pursue surrogacy or adoption due to religious, ethical, financial or legal constraints.^2 3^

## UTx from deceased donors—known ethical challenges

UTx from deceased donors represents a new intersection of assisted reproductive technology and organ transplantation. While UTx offers transformative possibilities, it also raises important ethical and social considerations. Key ethical arguments in favour of UTx from deceased donors include the avoidance of harm to living donors and the opportunity to provide fertility options to a greater number of women.^4^,^7^ However, there are also significant ethical concerns related to the procedure. These include the clinical risks to the recipient, the long-term medical and ethical uncertainties surrounding a relatively new procedure, allocation and the costs involved if UTx is offered through a public healthcare system.^4^,^7^

Additionally, complexities arise in the areas of consent and family decision-making.^4^,^7^ Most ethical assessments of uterus transplantation predominantly focus on the recipients, with less attention given to the deceased donors or their families. Although UTx from deceased donors is often viewed as morally preferable since it does not harm a living donor, the unique role of the uterus in creating life introduces complexity to the consent process. For instance, there are concerns about whether obtaining consent from family members is appropriate, given the organ’s reproductive purpose and the importance of individual procreative liberty.^5^

Currently, there is a scarcity of publications exploring the experiences of family members of UTx deceased donors, the benefits these family members may experience and their reasons for consenting to the procedure. As with other organ donations from deceased donors, potential benefits for donor families could be emotional or ethical, or provide a sense of solace. For instance, consenting to organ donation may help families find meaning after a tragic loss and receive societal recognition.^8 9^ However, for many families, complying with the donation wishes of deceased relatives can be difficult. One of the main reasons donations do not happen in practice is because of the refusal of family members.^10^ Given these complexities, further research on UTx from deceased donors is essential to better understand the perspectives of donor families and the factors influencing their decisions.

## Ethical challenges of UTx from deceased donors in a global health context

Despite its potential significance, the implications of this procedure, particularly the use of deceased women’s bodies—and especially in the Global South—have received limited international attention. UTx from deceased donors has been recognised as a remarkable advancement in fertility medicine.

However, ethical recommendations like those from the American Society for Reproductive Medicine position^11^ or The Montreal Protocol^12^—primarily reflect Western perspectives.^13^ These frameworks assume relatively high levels of gender equity, equality and robust healthcare systems, overlooking the unique ethical challenges that arise when UTx is performed in diverse socioeconomic contexts, particularly in low-resource settings.^13^

With the number of UTx procedures continuing to rise globally,^14^ there is a notable lack of research on how UTx from deceased donors might operate within a transnational context, both in clinical trials and outside those highly regulated settings. As the practice evolves, concerns have been raised about the potential rise of reproductive tourism, the phenomenon of people and technologies crossing international borders to access reproductive technologies. This trend risks exacerbating global inequalities as those with financial means gain access to reproductive options unavailable to local populations, all while relying on local healthcare systems and human resources.

Furthermore, the possibility of commercialising UTx from deceased donors could place undue pressure on families to donate organs and potential pressures on women to undergo reproductive interventions also arise.^13^ Although parenthood is a deeply valued aspiration for many—as indicated by the surrogacy market, estimated to be worth about US$14 billion in 2022^15^—the demand can produce unethical and exploitative practices, including child harvesting,^16^ baby factories^17^ and illegal surrogacy.^18^ Inherently, such practices highlight the commodification of the human body, particularly in societies where the burden of disadvantage often falls on women, especially those in vulnerable socioeconomic situations. These concerns are further exacerbated by the reality that fertility rates are declining globally, with the 15 countries that make up the largest economies by GDP (Gross Domestic Product) recording fertility rates below the replacement level, including many high-income countries, as well as China and India.^19^

Moreover, although UTx is classified as a life-enhancing rather than life-saving intervention, like face or hand transplants,^4^ the increasing demand for uteruses could inadvertently fuel organ trafficking, particularly given the global shortage of organs. While UTx aims to address uterine factor infertility, it operates within a broader context where reproductive technologies may be susceptible to abuse. This risk is heightened in regions with documented cases of organ trafficking,^20^ which often exploit vulnerable populations, such as refugees and impoverished communities, particularly in regions with significant sociopolitical instability.^21 22^

Furthermore, UTx is a novel procedure, and research suggests that preferences regarding tissue donation can be complex. Some individuals may be less willing to donate non-vital organs after death, with an even greater reluctance towards donating reproductive organs.^4^,^7^ Since UTx may be ethically contentious in certain societies and religious groups^23 24^ and is not widely known to the public, the level of acceptance for uterus donation is uncertain. The social and cultural implications of using a deceased person’s uterus for childbearing are not well understood, highlighting the need for careful considerations of different cultural contexts and further research and discussion in that area.

There are also concerns that requests for uterine donation could negatively impact consent rates for other life-saving organ donations. Issues like mistrust, lack of transparency or discomfort with the concept of reproductive organ donation can make families hesitant to approve any organ donation at all. As a result, obtaining consent from bereaved relatives for UTx may be challenging, potentially reducing the likelihood of obtaining consent for life-saving organ donations. However, to date, there have been no reports confirming these concerns regarding deceased donor UTx.^25^

## Conclusions

Given these competing considerations and uncertainties, there is a pressing need for empirical research and ethical analysis to enhance our understanding of the ethical dimensions of UTx from deceased donors in a global health context. Such an analysis is crucial for addressing questions such as the following: Which women count as important in the discussion of the solutions to Western infertility? Under what conditions is it acceptable to offer UTx from deceased donors? What safeguards are essential to protect the rights and interests of disadvantaged and marginalised women and their families? In examining these issues related to UTx from deceased donors, it is critical to consider whether this practice could unintentionally create new forms of exploitation or unethical reproductive tourism. Fuelling a shadow market for poor women’s uteruses could worsen existing inequalities and further marginalise vulnerable populations.

The enthusiasm surrounding this innovative technology should not overshadow the needs of some women while rendering the bodies and personhood of other deceased women ‘invisible’. This risk arises when ethical considerations fail to account for the diverse social, economic and geographical disparities among women. Collaborations between researchers and policy makers should prioritise the development of clear, globally inclusive guidelines that address the unique ethical challenges of UTx from deceased donors in various socioeconomic contexts. This is a potentially challenging space that will contribute to developing new knowledge and policy recommendations. Ensuring equitable access, protecting vulnerable populations and preventing exploitation must be at the forefront of these guidelines.

## Data Availability

There are no data in this work.
